# Comparison of the effect of phenobarbital & levetiracetam in the treatment of neonatal abstinence syndrome (NAS) as adjuvant treatment in neonates admitted to the neonatal intensive care unit: a randomized clinical trial

**DOI:** 10.1186/s12884-024-06433-y

**Published:** 2024-04-05

**Authors:** Zahra Jamali, Mohammad Hosein Molaei-Farsangi, Habibeh Ahmadipour, Bahareh Bahmanbijari, Fatemeh Sabzevari, Zahra Daei Parizi

**Affiliations:** 1 Department of Pediatrics, School of Medicine, Afzalipour Hospital, Kerman University of Medical Sciences, Kerman, Iran; 2 Department of Pediatrics, School of Medicine; Clinical Research Development Unit, Afzalipour Hospital, Kerman University of Medical Sciences, Kerman, Iran; 3 Department of Social Medicine, School of Medicine, Social Determinants of Health Research Center, Institute for Futures Studies in Health, Kerman University of Medical Sciences, Kerman, Iran; 4 Department of Pediatrics, School of Medicine, Kerman University of Medical Sciences, Kerman, Iran

**Keywords:** Neonatal abstinence syndrome, Phenobarbital, Levetiracetam, Alternative treatment

## Abstract

**Background:**

Infants who are born from mothers with substance use disorder might suffer from neonatal abstinence syndrome (NAS) and need treatment with medicines. One of these medicines is phenobarbital, which may cause side effects in long-term consumption. Alternative drugs can be used to reduce these side effects. This study seeks the comparison of the effects of phenobarbital & levetiracetam as adjuvant therapy in neonatal abstinence syndrome.

**Methods:**

This randomized clinical trial was performed in one year from May 2021 until May 2022. The neonates who were born from mothers with substance use disorder and had neonatal abstinence syndrome in Afzalipoor Hospital of Kerman were studied. The treatment started with morphine initially and every four hours the infants were checked. The infants who were diagnosed with uncontrolled symptoms After obtaining informed consent from the parents were randomly divided into two groups and treated with secondary drugs, either phenobarbital or levetiracetam.

**Results:**

Based on the obtained results, it was clear that there was no significant difference between the hospitalization time of the two infant groups under therapy (phenobarbital: 18.59 days versus Levetiracetam 18.24 days) (*P*-value = 0.512). Also, there was no significant difference between both groups in terms of the frequency of re-hospitalization during the first week after discharge, the occurrence of complications, and third treatment line prescription (*P*-value = 0.644).

**Conclusions:**

Based on the obtained results, like hospitalization duration time (*P*-value = 0.512) it seems that levetiracetam can be used to substitute phenobarbital in treating neonatal abstinence syndrome.

**Trial registration:**

The current study has been registered in the Iran registry of clinical trials website (fa.irct.ir) on the date 25/2/2022 with registration no. IRCT20211218053444N2.

**Supplementary Information:**

The online version contains supplementary material available at 10.1186/s12884-024-06433-y.

## Background

Pregnant women are considered an important group of opium users. All opioid drugs can pass through the placenta and reach the infant.so that neonates may suffer from neonatal abstinence syndrome (NAS) [1]. Likewise, in Iran, a number of pregnant women have substance use disorder, which affects their infants and may show signs of NAS afterward [2].

NAS is a complicated disorder that will affect the central autonomic nervous system and the digestive system in the first stage [3]. Symptoms of this syndrome vary from mild tremors to yawning, sneezing, extreme restlessness, fever, feeding intolerance and seizure [4]. The clinical manifestation of this syndrome usually appears in the first days after birth. The onset time and the severity of the drug withdrawal symptoms depend on numerous factors such as the drug type and the amount used by the mother, the last drug usage time before giving birth and simultaneous usage of other drugs like Benzodiazepine and Barbiturate drugs [5, 6].

Neonatal abstinence syndrome treatment starts with non-pharmacological support like placing the infant in a calm environment with low light, swaddling the infant, using a pacifier and feeding the infant adequately, specifically exclusive breastfeeding [6]. Non-pharmacological treatment, however, was not effective for almost 80% of infants, and pharmacological therapy was required to control the symptoms of their neonatal abstinence syndrome [7]. The American Academy of Pediatrics recommends using opioid drugs as the initial treatment of neonatal abstinence syndrome [6], but it has been suggested by various studies that combination treatments can be more effective than the treatment by opioid drug prescription alone [7].

Despite of the appropriate treatment of the infants having neonatal abstinence syndrome with opioid drugs, some infants might need secondary or adjuvant therapy [8]. Phenobarbital is the most common supplementary drug used for treating neonatal abstinence syndrome and it is most often used as an adjuvant treatment for those infants with neonatal abstinence syndrome whose mother are multi drug user [7]. However, there are concerns over long-term neuro-evolution and behavioral effects in infants under the treatment with phenobarbital and the side effects caused by this drug have not been widely studied in human research [9–11]. Long-term side effects such as learning difficulties, further language and cognitive disorders in future have been seen in infants who were under phenobarbital treatment [12]. Sleep disorder and aggressive behavior have been reported in some studies [13]. Mental performance difficulties and hyperactivity have also been observed in some studies performed on these children [14]. Considering this issue, an alternative medicine for phenobarbital is highly needed.

Phenobarbital works by activating the receptors of GABA (Gama-aminobutyric messenger), which is an inhibitory neurotransmitter that controls convulsions [15]. Based on this mechanism, other anticonvulsant drugs with the same mechanism but lesser side effects may be used to control symptoms of neonatal abstinence syndrome. One of these drugs is levetiracetam, which has lesser side effects and has relatively the same mechanism as phenobarbital. Therefore, the aim of this prospective study is to investigate and compare the effects of phenobarbital and levetiracetam as an adjuvant therapy in infants with neonatal abstinence syndrome whose symptoms could not be controlled by morphine alone.

## Methods

This randomized clinical trial was performed with an ethical code of IR.KMU.AH.REC.1400.165 obtained from the Ethics Committee of Kerman University of Medical Sciences. By obtaining written consent from the parents 82 infants suffering from neonatal abstinence syndrome who were hospitalized in neonatal intensive care unit (NICU ward) of the Afzalipoor hospital of Kerman between the first days of May 2021 to the first day of May 2022 were included in this study without blinding. And the duration of data collection lasted about one year. Sampling was done according to the entry and exit criteria and with regard to the study of Surran et al. [16].

### Study’s inclusion criteria

Hospitalized Infants with the gestational age of 35 weeks and more, suffering from neonatal abstinence syndrome.

### Study’s exclusion criteria

Infants with major congenital abnormality; infants with other reasons for hospitalization and infants whose parents did not consent for the study.

The first outcome of this study was the hospitalization duration after receiving the secondary adjuvant treatment, the second outcome was the frequency of re-hospitalization after receiving the second adjuvant treatment in the first week after discharge and the occurrence of complications such as seizure, feeding intolerance, diarrhea, bradycardia, oxygen desaturation, lethargy, poor feeding, hypothermia, emesis and the need for third adjuvant treatment.

The current study has been registered in the Iran registry of clinical trials website (fa.irct.ir) on the date 25/2/2022 with registration no. IRCT20211218053444N2.

The Finnegan score system was used to diagnose infants with neonatal abstinence syndrome in order to carry out the research. If an infant had the Finnegan score above 8 in 3 consecutive evaluations or the score above 12 in 2 consecutive evaluations, s/he would be hospitalized, and at first treated with morphine using the pediatrics appropriate dosage based on her/his score and would be visited by neonatologist or fellowship of neonatology every four hours to check the symptoms of abstinence syndrome like restlessness, tremor, yawning, etc. [15–17].

The infants, who were diagnosed with uncontrolled symptoms (with the maximum dose of morphine) by the specialist, were divided into two groups based on the random allocation rule; one group was treated with phenobarbital, and the other with levetiracetam as the secondary medicine. The maximum dosage amount of phenobarbital and levetiracetam given to the infants based on the morphine dosages as the initial therapy and the Finnegan score is demonstrated in the Table 1. The dosage of levetiracetam was adjusted based on the anticonvulsant dose of the drug in infants.

**Table 1:** The received dose of phenobarbital and levetiracetam [15–18]

Finnegan score	The initial dose of Tincture of morphine	Phenobarbital	Levetiracetam
8–10	0.25-0.4 mg /kg/day Divided q 4 h	5 mg /kg day Divided q 12 h	30 mg/kg/day Divided q 12 h
11–13	0.45-0.6 mg /kg/day Divided q 4 h	7 mg /kg /day Divided q 12 h	40 mg/kg/day Divided q 12 h
14–16	0.65-0.8 mg /kg/day Divided q 4 h	10 mg/kg /day Divided q12 hours	50 mg/kg/day Divided q 12 h
17 and above	0.85-1.2 mg /kg/day Divided q 4 h	12 mg/kg/day Divided q 12 h	60 mg /kg/day Divided q 12 h

Infant’s characteristics such as gestational age, birth weight, hospitalization duration, frequency of re-hospitalization, occurrence of complications like seizure, and the need to receive secondary adjuvant medicine were registered in the data registration form. Medical interview, examination and case history results were registered in the form to complement the information needed. The drug withdrawal symptoms of infants based on the Finnegan score system [17] was also registered in the data registration form.

Data analysis was done using SPSS software version no. 24. Descriptive statistics measures, frequency, frequency percentage, average and standard deviation and Chi-square and Mann-Whitney U statistical tests were used to analyze data.

## Results

During the study, 108 neonates with abstinence syndrome were admitted in the neonatal intensive care unit, 5 cases who suffered from congenital anomalies and 6 cases who were born with the gestational age of less than 35 weeks and 15 cases due to lack of parental consent were not included in the study eventually. Eighty-two infants were included in this study; 41 infants were placed in the phenobarbital treatment group, and 41 others in the levetiracetam treatment group. The qualitative and quantitative demographic data are shown in the Table 2.

**Table 2:** The qualitative and quantitative demographic data of the research participants

Variable	Phenobarbital treatment group		Levetiracetam treatment group		*P*-value
	Frequency	Percentage	Frequency	Percentage	
**Sex**					
Male	23	56.1	24	58.5	0.823
Female	18	43.9	17	41.5	
**Mothers age at the time of childbirth**					
25 years and lower	11	26.8	6	14.6	0.189
26–30 years	8	19.5	13	31.7	
31–35 years	11	26.8	11	26.8	
36–40 years	11	26.8	8	19.5	
Above 40 years	0	0.0	3	7.3	
**Route of delivery**					
Vaginal delivery	19	46.3	14	34.1	0.260
Cesarean section	22	53.7	27	65.9	
**Mother’s addiction type**					
Opium	19	46.3	22	53.7	0.236
Heroin & opium	6	14.6	4	9.8	
Heroin	2	4.9	2	4.9	
Methadone	8	19.5	4	9.8	
Buprenorphine	1	2.4	0	0.0	
Crystal & opium	2	4.9	0	0.0	
Opium juice & methadone	2	4.9	0	0.0	
Methadone & heroin	1	2.4	3	7.3	
Crystal & methadone	0	0.0	2	4.9	
Heroin & crystal	0	0.0	1	2.4	
Opium juice	0	0.0	2	4.9	
Methadone, heroin & crystal	0	0.0	1	2.4	
**Mother’s addiction time**					
Lower than 12 months	1	2.4	2	4.9	0.324
12–24 months	19	46.3	23	56.1	
24–36 months	7	17.1	7	17.1	
36–48 months	5	12.2	7	17.1	
48–60 months	7	17.1	2	4.9	
Above 60 months	2	4.9	0	0.0	
**Gestational age (week) (mean + SD)**	38.29	1.48	38.15	1.52	0.152
**Finnegan score (mean + SD)**	10.61	1.27	10.85	1.15	0.301
**Weight at birth (gram) (mean + SD)**	2733.90	344.15	2729.27	389.38	0.830

Based on Table 3, although the hospitalization duration of the levetiracetam group is slightly lesser than phenobarbital group, this difference is not meaningful statistically (*p*-value = 0.512) and so, there was no meaningful statistical difference between the two phenobarbital and levetiracetam groups in the average of hospitalization time.

**Table 3:** The results of comparing the effects of Phenobarbital and Levetiracetam on the condition of infants

	Phenobarbital		Levetiracetam		*P*-value
	Mean	Standard deviation	Mean	Standard deviation	
**Hospitalization time (per day)**	18.59	1.87	18.24	1.69	0.512
**Rehospitalization**					
yes	0	0.0	0	0.0	-
no	41	100.0	41	100.0	
**Side effect**					
Positive	0	0.0	0	0.0	-
Negative	41	100.0	41	100.0	
**Tertiary Therapy**					
Positive	2	4.9	3	7.3	0.644
Negative	39	95.1	38	92.7	

None of the groups needed re-hospitalization. So, there was no difference between the two groups in the frequency of re-hospitalization.

There was no special complication manifested by any infant in both groups under treatment, therefore, there was no significant difference between the two groups in the frequency of occurrence of complications.

In this study, when the symptoms were not controlled with the maximum dose of the second drug a third drug which was clonidine was considered. It was manifested that two infants in the group under phenobarbital treatment needed the third treatment line, while the number of infants that needed the third treatment line was three in the group under levetiracetam treatment. However, there was no significant statistical difference between the two groups in this frequency (*p*-value = 0.644).

From the secondary results of this study, it was found that the type of mother’s addiction and the duration of her addiction had no relationship with the incidence of complications occurrence, re-hospitalization, and the need of third treatment.

## Discussion

This study was conducted with the aim of comparing the effect of adjuvant treatment with phenobarbital and levetiracetam in infants with neonatal abstinence syndrome who were hospitalized in the NICU. Results obtained from the data analysis of this study showed that hospitalization duration of the group that was under levetiracetam treatment was slightly lesser than that of the group under phenobarbital treatment, although this reduction was not statistically meaningful. Also, it was manifested that none of the groups needed re-hospitalization, also, there were no signs of complications among the infants and only two infants in the phenobarbital group and three infants in the levetiracetam group needed third-line adjuvant treatment in which there was also no significant difference in this frequency between the two groups. Therefore, using levetiracetam has the same effect as phenobarbital in reducing neonatal abstinence syndrome; in a way, that there was no need to hospitalize the infants again and there were no signs of complications that required medical care.

Regarding the effectiveness of phenobarbital and levetiracetam drugs in treating seizure, it was shown in the studies conducted on the treatment of seizure among the infants that using phenobarbital drug leads to side effects such as hypotension, hypoventilation [19–21], abnormal heart rate and hypothermia [22, 23] in some infants, while these side effects frequency is much lower in the groups who go under the treatment of levetiracetam. Also, using phenobarbital may harm the brain under growth, accelerate the apoptosis of nerve cells and ultimately, lead to cognitive disorders [24], but using levetiracetam, which is recently used as the anticonvulsant treatment, has more advantages such as fewer side effects and fewer nerve injuries of the infants. In such a way, that serious side effects manifestation like hypotension in patients under levetiracetam treatment is much lesser than that of patients under phenobarbital treatment. Therefore, it is possible to use this drug as a substitute to phenobarbital.

In studies related to the treatment of neonatal abstinence syndrome under pharmacological therapy, limited cases of that treated by phenobarbital drug have been mentioned in which the effectiveness of this drug has been confirmed. In the research of Merhar et al. in 2021, it was declared that among the infants with neonatal abstinence syndrome, those who had been under phenobarbital treatment had lesser hospitalization duration and faster treatment in comparison to the group under clonidine treatment. The hospitalization duration of the phenobarbital group was averagely 26.4 days and for clonidine was averagely 36.5 days [25]. Regarding this matter, the hospitalization days’ count in the aforementioned study is different from ours in both groups under treatments of phenobarbital and levetiracetam.

Brusseau et al. research in 2020, also, concluded that after comparing the effectiveness of phenobarbital and clonidine as an adjuvant treatment in treating neonatal abstinence syndrome, the average treatment time of clonidine (34.4 days) was significantly higher than that of phenobarbital (25.5 days). In the group that was under clonidine treatment, infants’ hospitalization duration was also much more than phenobarbital for adjuvant treatment (33.8 days against of 22 days). Thus, phenobarbital as an adjuvant treatment had a shorter hospitalization duration. Also, there was no sign of side effects in the phenobarbital group of infants, however, six infants in the clonidine group showed side effects [26]. In our current study, the average number of hospitalization days of phenobarbital was equal to 17.59 days and of levetiracetam was equal to 17.24, which is shorter than the previous studies. Regarding the side effects, also, no infant neither in the phenobarbital nor levetiracetam groups have shown any special side effects.

Nayeri et al. compared the effectiveness of phenobarbital and morphine in controlling the neonatal abstinence syndrome in a randomized clinical trial in 2015 and concluded that there was no significant difference in the duration of the treatment, hospitalization time and the need to receive adjuvant therapy between infants with neonatal abstinence syndrome treatment who received phenobarbital treatment and those who received morphine, therefore, they concluded that phenobarbital can be used as an effective treatment in reducing neonatal abstinence syndrome symptoms [27]. Based on the results obtained from our research, there was a significant difference between phenobarbital and levetiracetam groups in the average hospitalization duration compared to the previous study, also in our study, the hospitalization days was significantly higher, however, regarding the side effects manifested by the infants in the previous study was similar to ours.

The conclusion of the Surran et al. research in 2013 indicated that phenobarbital, as an adjuvant treatment would clinically shorten the hospitalization time (12.4 days in phenobarbital & 19.5 in the clonidine group on average) [16]. The result of this study in the phenobarbital group was almost similar to our study in terms of hospitalization days’ count.

Zimmermann et al. compared the effectiveness of phenobarbital, morphine and chlorpromazine in treating neonatal abstinence syndrome. In that study, the average duration of treatment for morphine was 22 days, chlorpromazine was 25 days, and phenobarbital was 32 days [28]. In our study, the duration of treatment in both phenobarbital and levetiracetam groups was less than the mentioned study groups.

Several factors can affect the differences created between the results of the present study and other studies: one of these factors can be the difference in variables such as race and ethnicity, and the amount of opioid consumption of the mother. Another factor that can be important in creating this difference is the types of opioid substances that mothers abused during pregnancy. Example, in Nayeri et al. study, cocaine was the most abused substance drug by mothers in the phenobarbital treatment group. Also, In Brusseau et al. study, the most abused substance drug by mothers was Buprenorphine. While in the current study, the most abused substance drug was related to opium. Also, another affecting factor can be scoring systems and the study entry criteria differences.

This study includes a small group of infants with this syndrome. However, it is recommended to perform similar studies with larger groups in different provinces of Iran and worldwide to prove this theory. Also, infants should be observed and followed up in long terms in order to make clear that there won’t be any side effects for them when they grew older. If the current conclusion is proven in other researches, levetiracetam can be regarded as an alternative secondary therapy of neonatal abstinence syndrome.

## Conclusions

The results of the current study confirmed the efficiency of prescribing levetiracetam as a non-opioid drug in treating neonatal abstinence syndrome compared to phenobarbital. This efficiency has been present in hospitalization days’ count, the lack of side effects, and the independency toward re-hospitalization. Therefore, it seems that levetiracetam can be used as an alternative treatment to phenobarbital.

### Electronic supplementary material

Below is the link to the electronic supplementary material.

Supplementary Material 1

Supplementary Material 2

## Data Availability

All data generated or analyzed during this study are included in this published article.
