# Supplementary material for: Comparison of the effect of phenobarbital & levetiracetam in the treatment of neonatal abstinence syndrome (NAS) as adjuvant treatment in neonates admitted to the neonatal intensive care unit: a randomized clinical trial
Source: BMC Pregnancy Childbirth. 2024 Apr 5;24:242. doi: 10.1186/s12884-024-06433-y (PMC10996075; doi:10.1186/s12884-024-06433-y)
Supplement: Supplementary file 1 — Supplementary Material 1 [file 12884_2024_6433_MOESM1_ESM.docx]

**Research protocol: part 1**

**Project summary**

Background: Infants who are born from mothers with substance use disorder might suffer from neonatal abstinence syndrome (NAS) and need treatment. If the initial line therapies are not effective, secondary line therapies will be used. One of these medicines is phenobarbital, which may cause side effects in long-term consumption. Alternative drugs can be used to reduce these side effects. This study seeks the comparison of the effects of phenobarbital & levetiracetam as adjuvant therapy in neonatal abstinence syndrome.

Methods: This randomized clinical trial was performed in one year from May 2021 until May 2022. The neonates who were born from mothers with substance use disorder and had neonatal abstinence syndrome in Afzalipoor Hospital of Kerman were studied. The treatment started with morphine initially and every four hours the infants were checked. The infants who were diagnosed with uncontrolled symptoms were randomly divided into two groups and treated with secondary drugs, either phenobarbital or levetiracetam. The data were registered in the data registration forms and analyzed with the SPSS software.

At the beginning of this study, we expected that levetiracetam, which has fewer side effects than phenobarbital, would be at least as effective as phenobarbital in the adjunctive treatment of neonatal abstinence syndrome.

**General information**

**Neonatal abstinence syndrome**

Sponsor: Kerman University of Medical Sciences

Zahra Jamali Assistant Professor of Neonatal-Perinatal Medicine, Department of Pediatrics, School of Medicine, Afzalipour Hospital, Kerman University of Medical Sciences, Kerman, Iran

Site of research: Afzalipour Hospital in Kerman

**Rationale & background information**

Phenobarbital is the most common supplementary drug used for treating neonatal abstinence syndrome and it is most often used as an adjuvant treatment for those infants with neonatal abstinence syndrome whose mother are multi drug user. However, there are concerns over long-term neuro-evolution and behavioral effects in infants under the treatment with phenobarbital and the side effects caused by this drug have not been widely studied in human research. Long-term side effects such as learning difficulties, further language, and cognitive disorders in future have been seen in infants who were under phenobarbital treatment. Sleep disorder and aggressive behavior have been reported in some studies. Mental performance difficulties and hyperactivity have also been observed in some studies performed on these children. Considering this issue, an alternative medicine for phenobarbital is highly needed.

Although, in recent years, many researches have been conducted in the field of this syndrome but there are still many shortcomings in the management of this syndrome.

Phenobarbital works by activating the receptors of GABA (Gama-aminobutyric messenger), which is an inhibitory neurotransmitter that controls convulsions (15). Based on this mechanism, other anticonvulsant drugs with the same mechanism but lesser side effects may be used to control symptoms of neonatal abstinence syndrome. One of these drugs is levetiracetam, which has lesser side effects and has relatively the same mechanism as phenobarbital. Therefore, the aim of this prospective study is to investigate and compare the effects of phenobarbital and levetiracetam as an adjuvant therapy in infants with neonatal abstinence syndrome whose symptoms could not be controlled by morphine alone.

**References**

1. Griffiths SK, Campbell JP. Placental structure, function and drug transfer. Continuing Education in Anaesthesia, Critical Care & Pain. 2015;15(2):84-9.

2. Mashmool A, Bakhshani N. Prevalence of drug withdrawal symptoms in infants and comparison symptoms according to mothers job and infants birth order in Zahedan. Journal of Research in Behavioural Sciences. 2015;13(1):22-8. [In Persian].

3. Patrick SW, Schumacher RE, Benneyworth BD, Krans EE, McAllister JM, Davis MM. Neonatal abstinence syndrome and associated health care expenditures: United states, 2000-2009. JAMA. 2012;307(18):1934-40.

4. Bio LL, Siu A, Poon CY. Update on the pharmacologic management of neonatal abstinence syndrome. Journal of perinatology: official journal of the California Perinatal Association. 2011;31(11):692-701.

5. Kocherlakota P. Neonatal abstinence syndrome. Pediatrics. 2014;134(2):e547-e61.

6. Hudak M, Tan R. Committee on drugs; committee on fetus and newborn; American academy of pediatrics. Neonatal drug withdrawal Pediatrics. 2012;129(2):e540-e60.

7. Coyle MG, Ferguson A, Lagasse L, Oh W, Lester B. Diluted tincture of opium (dto) and phenobarbital versus dto alone for neonatal opiate withdrawal in term infants. The Journal of pediatrics. 2002;140(5):561-4.

8. Agthe AG, Kim GR, Mathias KB, et al. Clonidine as an adjunct therapy to opioids for neonatal abstinence syndrome: A randomized, controlled trial. Pediatrics. 2009;123(5):e849-56.

9. Chen J, Cai F, Cao J, Zhang X, Li S. Long-term antiepileptic drug administration during early life inhibits hippocampal neurogenesis in the developing brain. Journal of neuroscience research. 2009;87(13):2898-907.

10. Meador KJ, Baker G, Cohen MJ, Gaily E, Westerveld M. Cognitive/behavioral teratogenetic effects of antiepileptic drugs. Epilepsy & behavior: E&B. 2007;11(3):292-302.

11. Bhardwaj SK, Forcelli PA, Palchik G, Gale K, Srivastava LK, Kondratyev A. Neonatal exposure to phenobarbital potentiates schizophrenia-like behavioral outcomes in the rat. Neuropharmacology. 2012;62(7):2337-45.

12. Sulzbacher S, Farwell JR, Temkin N, Lu AS, Hirtz DG. Late cognitive effects of early treatment with phenobarbital. Clinical pediatrics. 1999;38(7):387-94.

13. Camfield CS, Chaplin S, Doyle AB, Shapiro SH, Cummings C, Camfield PR. Side effects of phenobarbital in toddlers; behavioral and cognitive aspects. The Journal of pediatrics. 1979;95(3):361-5.

14. Vining EP, Mellitis ED, Dorsen MM, et al. Psychologic and behavioral effects of antiepileptic drugs in children: A double-blind comparison between phenobarbital and valproic acid. Pediatrics. 1987;80(2):165-74.

15. Swaiman KF, Ashwal S, Ferriero DM, et al. Swaiman's pediatric neurology: Principles and practice. 6th ed. Amsterdam: Elsevier Health Sciences; 2017.

16. Surran B, Visintainer P, Chamberlain S, Kopcza K, Shah B, Singh R. Efficacy of clonidine versus phenobarbital in reducing neonatal morphine sulfate therapy days for neonatal abstinence syndrome. A prospective randomized clinical trial. Journal of perinatology: official journal of the California Perinatal Association. 2013;33(12):954-9.

17. Martin RJ, Fanaroff AA, Walsh MC. Fanaroff and martin's neonatal-perinatal medicine e-book: Diseases of the fetus and infant: Elsevier Health Sciences; 2020.

18. Lissauer T, Fanaroff AA, Miall L, Fanaroff J. Neonatology at a glance. New York: John Wiley & Sons; 2020.

19. Sharpe C, Reiner GE, Davis SL, et al. Levetiracetam versus phenobarbital for neonatal seizures: A randomized controlled trial. Pediatrics. 2020;145(6).

20. Thibault C, Naim MY, Abend NS, et al. A retrospective comparison of phenobarbital and levetiracetam for the treatment of seizures following cardiac surgery in neonates. Epilepsia. 2020;61(4):627-35.

21. Gowda VK, Romana A, Shivanna NH, Benakappa N, Benakappa A. Levetiracetam versus phenobarbitone in neonatal seizures - a randomized controlled trial. Indian pediatrics. 2019;56(8):643-6.

22. El-Dib M, Soul JS. The use of phenobarbital and other anti-seizure drugs in newborns. Seminars in fetal & neonatal medicine. 2017;22(5):321-7.

23. Soul JS, Bergin AM, Stopp C, et al. A pilot randomized, controlled, double-blind trial of bumetanide to treat neonatal seizures. Annals of neurology. 2021;89(2):327-40.

24. Qiao MY, Cui HT, Zhao LZ, Miao JK, Chen QX. Efficacy and safety of levetiracetam vs. Phenobarbital for neonatal seizures: A systematic review and meta-analysis. Frontiers in neurology. 2021;12:747745.

25. Merhar SL, Ounpraseuth S, Devlin LA, et al. Phenobarbital and clonidine as secondary medications for neonatal opioid withdrawal syndrome. Pediatrics. 2021;147(3).

26. Brusseau C, Burnette T, Heidel RE. Clonidine versus phenobarbital as adjunctive therapy for neonatal abstinence syndrome. Journal of perinatology: official journal of the California Perinatal Association. 2020;40(7):1050-5.

27. Nayeri F, Sheikh M, Kalani M, et al. Phenobarbital versus morphine in the management of neonatal abstinence syndrome, a randomized control trial. BMC pediatrics. 2015;15:57.

28. Zimmermann U, Christoph R, Angelo D, et al. Treatment of opioid withdrawal in neonates with morphine, phenobarbital, or chlorpromazine: a randomized double-blind trial. European Journal of Pediatrics. 2020;179: 141–9.

**Study goals and objectives**

**Primary objective**: Comparison of the effect of phenobarbital & levetiracetam in the treatment of neonatal abstinence syndrome (NAS) as adjuvant treatment in neonates admitted to the neonatal intensive care unit

**Secondary goals:**

1: Determination of the effect of levetiracetam in reducing the duration of morphine consumption in neonatal abstinence syndrome compared to phenobarbital

2: Determining the effect of adjuvant treatment with levetiracetam in reducing the dose of morphine in neonatal abstinence syndrome compared to phenobarbital

3: Determining the duration of hospitalization in neonatal abstinence syndrome in adjuvant treatment with levetiracetam compared to phenobarbital

4: Determining the readmission rate during the first week after discharge in the adjunctive treatment with levetiracetam compared to phenobarbital

5: Determining the incidence of side effects such as seizures in adjuvant treatment with levetiracetam compared to phenobarbital

6: Determining the number of infants requiring third adjuvant treatment in neonatal abstinence syndrome in adjuvant treatment with levetiracetam compared to phenobarbital

**Study design**

This randomized clinical trial study was a prospective case-control study.

During the study, 108 neonates with abstinence syndrome were admitted in the neonatal intensive care unit, 5 cases who suffered from congenital anomalies and 6 cases who were born with the gestational age of less than 35 weeks and 15 cases due to lack of parental consent were not included in the study eventually. By obtaining written consent from the parents’ 82 infants suffering from neonatal abstinence syndrome who were hospitalized in NICU ward of the Afzalipour hospital of Kerman between 2021 to 2022 were included in this study without blinding. Sampling was done according to the entry and exit criteria and with regard to the study of Surran et al. (16).

**Study’s inclusion criteria:** Hospitalized Infants with the gestational age of 35 weeks and more, suffering from neonatal abstinence syndrome.

**Study’s exclusion criteria:** Infants with the gestational age of less than 35 weeks; infants with major congenital abnormality; infants with other reasons for hospitalization and infants whose parents did not consent for the study.

**Methodology**

This study was performed in the NICU ward of Afzalipoor hospital of Kerman. The first outcome of this study was the hospitalization duration after receiving the secondary adjuvant treatment, the second outcome was the frequency of re-hospitalization after receiving the second adjuvant treatment in the first week after discharge and the occurrence of complications such as seizure, feeding intolerance, diarrhea, bradycardia, oxygen desaturation, lethargy, poor feeding, hypothermia, emesis and the need for third adjuvant treatment.

The current study has been registered in the Iran registry of clinical trials website (fa.irct.ir) on the date 25/2/2022 with registration no. IRCT20211218053444N2.

The Finnegan score system was used to diagnose infants with neonatal abstinence syndrome in order to carry out the research. If an infant had the Finnegan score above 8 in 3 consecutive evaluations or the score above 12 in 2 consecutive evaluations, s/he would be hospitalized, and at first treated with morphine using the pediatrics appropriate dosage based on her/his score and would be visited by neonatologist or fellowship of neonatology every four hours to check the symptoms of abstinence syndrome like restlessness, tremor, yawning, etc.

The infants, who were diagnosed with uncontrolled symptoms (with the maximum dose of morphine) by the specialist, were divided into two groups based on the random allocation rule; one group was treated with phenobarbital, and the other with levetiracetam as the secondary medicine. This study was an unblinded study. The maximum dosage amount of phenobarbital and levetiracetam given to the infants based on the morphine dosages as the initial therapy and the Finnegan score is demonstrated in the tables 1. The dosage of levetiracetam was adjusted based on the anticonvulsant dose of the drug in infants. Infant’s characteristics such as gestational age, birth weight, hospitalization duration, frequency of re-hospitalization, occurrence of complications like seizure, and the need to receive secondary adjuvant medicine were registered in the data registration form. Medical interview, examination and case history results were registered in the form to complement the information needed. The drug withdrawal symptoms of infants based on the Finnegan score system was also registered in the data registration form.

Table 1. The received dose of phenobarbital and levetiracetam (15-18).

| **Finnegan score** | **The initial dose of Tincture of morphine** | **Phenobarbital** | **Levetiracetam** |
| --- | --- | --- | --- |
| 8-10 | 0.25-0.4mg /kg/day  Divided q 4 hours | 5 mg /kg day  Divided q 12 hours | 30 mg/kg/day  Divided q 12 hours |
| 11-13 | 0.45-0.6mg /kg/day  Divided q 4 hours | 7 mg /kg /day  Divided q 12 hours | 40 mg/kg/day  Divided q 12 hours |
| 14-16 | 0.65-0.8mg /kg/day  Divided q 4 hours | 10 mg/kg /day  Divided q12 hours | 50 mg/kg/day  Divided q 12 hours |
| 17 and above | 0.85-1.2mg /kg/day  Divided q 4 hours | 12 mg/kg/day  Divided q 12 hours | 60 mg /kg/day  Divided q 12 hours |

**Safety considerations**

If an unpredictable complication occurred at any stage of the study in the case group, the person was excluded from the study and the previous standard treatment was used.

**Follow-up**

Our team followed up the neonates for complications through phone calls for two weeks after discharge.

**Data management and statistical analysis**

The sample size was calculated based on the study of Surran et al. using JPower software, 34 people in each group.

**T tests -** Means: Difference between two independent means (two groups)

**Analysis:** A priori: Compute required sample size

**Input:** Tail(s) = Two

Effect size d = 0.7

α err prob = 0.05

Power (1-β err prob) = 0.8

Allocation ratio N2/N1 = 1

**Output:** Noncentrality parameter δ = 2.8861739

Critical t = 1.9965644

Df = 66

**Sample size group 1 = 34**

**Sample size group 2 = 34**

**Total sample size = 68**

Infants' characteristics such as gestational age and birth weight, hospitalization time, frequency of re-hospitalization, incidence of complications such as convulsions, and the need for third Adjuvant drugs were recorded in the data registration form.

A questionnaire form was prepared for each patient. To complete the necessary information in each form, the results of the interview and examination and history were used.

Data analysis was done using SPSS version 26 software. Descriptive statistics indicators of frequency, frequency percentage, mean and standard deviation and Chi-square and U-Man-Whitney statistical tests were used for data analysis.

**Quality assurance**

Our team followed up the neonates for complications through phone calls for two weeks after discharge.

**Expected outcomes of the study**

This study was conducted based on the hypothesis that adjunctive treatment with levetiracetam in neonatal deprivation syndrome can have fewer complications with an effect at least equal to that of phenobarbital. Therefore, proving this hypothesis can be of great help in improving the prognosis of these babies in the future and have positive effects on the health system of society.

**Dissemination of results and publication policy**

After conducting the study, the corresponding author will publish the results in the form of a scientific article in a reliable journal.

**Duration of the project**

Proposal preparation: 1 month

Approval of the proposal: 1 month

Collecting data: 12 months

Statistical analysis: 2 months

Writing an article: 2 months

**Problems anticipated**

Taking into account that the consent of the parents to leave the hospital happened before the end of the treatment period, the collection of samples and the work progressed slowly.

Some parents also did not accept the randomized conditions which was attempted by talking to them and explaining the benefits of the treatment method and satisfied them to participate in the study.

**Project management**

Zahra Jamali: data collecting

Mohammadhosein Molaei: data collecting

Habibeh Ahmadipour: data analysis

Bahareh Bahmanbijari: data collecting

Fatemeh Sabzevari: data collecting

Zahra Daei Parizi: data collecting

**Ethics**

1. Obtaining permission from the Ethics Committee of Kerman University of Medical Sciences (code of ethics: IR.KMU.AH.REC.1400.165)

2. Registration of the proposal in the clinical trial system of Iran (Clinical trial ID: 61813)

3. Use of parental informed consent for all infants participating in the study

4. Anonymity of samples and confidentiality of information of all people participating in the study

5. Being obliged to preserve the completeness and scientific integrity of her research and adherence to ethical principles in designing, implementing and publishing the results.

**Informed consent forms**

Dear parents, this study aims to investigate the effect of levetiracetam drug in the treatment of neonatal abstinence syndrome, which mothers take opium during pregnancy.

Currently, phenobarbital is widely used to treat infantile abstinence syndrome, but this drug can cause some developmental complications in the future for these babies. Levetiracetam is a drug with a similar mechanism of action and much less harmful effects than phenobarbital, which is used in the treatment of seizures. Therefore, we assume that the use of levetiracetam in the Adjuvant treatment of neonatal abstinence syndrome can reduce the developmental complications of these infants in the future. If you intend to withdraw from the study at any stage, you can withdraw from the study. If there is a possibility of complications in your baby at any stage of the study, the infant will be immediately excluded from the study.
At this time, you are fully informed that your neonate participates in this study

**Budget**

The present study did not require a special budget.

**Other support for the project**

N/A.

**Curriculum Vitae of investigators**

Zahra Jamali :Assistant Professor of Neonatal-Perinatal Medicine, Department of Pediatrics, School of Medicine, Afzalipour Hospital, Kerman University of Medical Sciences, Kerman, Iran.

Mohammadhosein Molaei: Assistant Professor of Neonatal-Perinatal Medicine, Department of Pediatrics, School of Medicine; Clinical Research Development Unit, Afzalipour Hospital, Kerman University of Medical Sciences, Kerman, Iran.

Habibeh Ahmadipour: Associate Professor of Community Medicine, Department of Social Medicine, School of Medicine, Social Determinants of Health Research Center, Institute for Futures Studies in Health, Kerman University of Medical Sciences, Kerman, Iran.

Bahareh Bahmanbijari: Associate Professor of Neonatal-Perinatal Medicine, Department of Pediatrics, School of Medicine, Afzalipour Hospital, Kerman University of Medical Sciences, Kerman, Iran.

Fatemeh Sabzevari: Assistant Professor of Neonatal-Perinatal Medicine, Department of Pediatrics, School of Medicine, Afzalipour Hospital, Kerman University of Medical Sciences, Kerman, Iran.

Zahra Daei Parizi: Assistant Professor of Neonatal-Perinatal Medicine, Department of Pediatrics, School of Medicine, Kerman University of Medical Sciences, Kerman, Iran.
